# Pacing Strategy Models in 1500 m Male Freestyle Long-Course Swimming on the Basis of the All-Time Ranking

**DOI:** 10.3390/ijerph20064809

**Published:** 2023-03-09

**Authors:** Maciej Hołub, Arkadiusz Prajzner, Arkadiusz Stanula

**Affiliations:** 1 Laboratory of Performance Analysis in Sport, Institute of Sport Sciences, Academy of Physical Education in Katowice, 40-065 Katowice, Poland; m.holub@awf.katowice.pl; 2 Center for Neurocognitive Functions and Self-Regulation Research, Institute of Psychology, Pedagogical University of Krakow, 30-084 Krakow, Poland; arkadiusz.prajzner@up.krakow.pl

**Keywords:** pacing, strategy, split, freestyle, swimming

## Abstract

In long-distance swimming competitions, a pacing strategy is of considerable importance for the final result and for sporting success. The paper presents the pacing strategy models of the all-time best competitors in 1500 m male freestyle long-course swimming. The top 60 scores were retrieved from official websites. The results were divided into six groups of ten swim times each, with splits of 15 × 100, 5 × 300, 3 × 500, and 2 × 750 m, and then analysis of variance was used. The main effects of the competitor group order revealed with the analysis of variance were statistically significant (*p* < 0.001). The group effect size turned out very high (η_p_^2^ = 0.95). Consecutive groups of competitors achieved significantly slower results. The magnitude of the interaction effects of the competitor group order and distance splits was moderate (η_p_^2^ of 0.05–0.09) and statistically not significant. The main effects of the 3 × 500, 5 × 300, and 15 × 100 m splits were high and very high (η_p_^2^ of 0.33–0.75) and statistically significant (*p* < 0.001). The difference between the 2 × 750 m split was statistically not significant. The achieved values of the main effects led to the following trend. In the distance split, the first and last sections did not significantly differ from each other, nor did the middle sections. However, when the middle sections and the first and last sections were compared against each other, a significant discrepancy was observed. The pacing strategies of the best athletes in the history of the competition follow a very similar parabolic trend.

## 1. Introduction

In swimming competitions, as in numerous sports disciplines such as running or cycling, the pacing strategy has a substantial impact on the final time of the race and thus on the result and sporting success [1]. However, swimming, physiologically and mechanically, is quite different from other endurance sports, combining exertion-induced hyperpnea, whole-body immersion, and demanding swimming techniques [2]. Pacing strategy is a manner of distributing work and energy expenditure during a task [3,4], and it is a combination of anticipation, feedback, and the sum of the player’s previous experience [5]. Some authors claim that the pace distribution strategy pattern is stored in the swimmer’s long-term memory and used as a pattern in similar competitions in the near future [6,7]. Long-distance competitions, such as 1500 m freestyle swimming, are part of the Olympic programme [8], and the strategy and consistency in splitting the pace are essential due to the large number of laps swum and the long duration of the race [9]. The aim of the swimmer at this distance, the longest one that is held in the swimming pool, is to maintain the highest possible speed and finish the race in the shortest possible time [10]. There are also many other factors that influence the competition result, such as the length and frequency of the cycle, which strongly determine the athlete’s speed [11]. The arm and leg rhythm adopted by competitors also contributes to sporting success in long-distance swimming. Sanders and Psycharakis (2009) define this component as the basic swimming characteristic, which helps divide more efficient swimmers from less efficient ones [12]. There are many strategies for racing at long distances. Among the most commonly applied is the parabolic strategy, which involves starting the race at a higher speed, slowing down in the middle part of the distance, and increasing the pace again at the end [13]. Common pacing strategies also include the positive strategy of covering the first half of the distance at a faster pace and the negative strategy of starting the race with a lower speed and covering the second half of the distance at a faster pace [14]. In turn, the all-out strategy consists in performing maximum work from the start of the distance. There is also a strategy of keeping even pace over the entire distance, as well as variations in the parabolic strategy, such as the J-shaped strategy, in which competitors cover the initial section at a faster pace and then switch to an even speed up until the end of the race, or after covering most of the distance at an even pace, they complete the finishing section at a faster pace [4,15,16].

Athletes specializing in long distances swim more kilometres while training and are therefore susceptible to overtraining syndrome (OTS). OTS may be caused by systemic inflammation and subsequent effects on the central nervous system, including depressed mood, central fatigue, and resultant neurohormonal changes [17]. It is crucial that, in addition to the volume of kilometres and a well-developed training plan, they can take advantage of the opportunities provided by analysing the performance of top competitors and drawing conclusions that can be incorporated into their training. Currently, results that help create pacing strategy models based on the best achievements in the history of the competition are available. These models not only help outline some trends that athletes and their coaching staffs can pursue but also provide information on what practices should be avoided.

This paper presents an analysis of pacing strategies applied by all-time best competitors in 1500 m male freestyle long-course (50 m) swimming. They were divided into groups, and overall group trends in pacing strategies were determined as inferred from the analysis. It was examined whether there were differences between successive groups and ranked athletes, as well as whether any changes occurred between them in the distribution of pacing and in the race execution strategy. The general trend of the pacing strategy and the potential significant differences in the splits were also investigated.

## 2. Materials and Methods

### 2.1. Subjects

The 60 all-time best results achieved in 1500 m male freestyle long-course (50 m) swimming in accordance with the official ranking were retrieved from the www.fina.org and www.swimrankings.net websites (as of 26 June 2022). The results that did not involve splits were excluded and replaced with subsequent ones from the list. Ultimately, 79 competitors were considered, of whom 19 were excluded due to the lack of detailed data. The scores were converted to seconds and then divided into 6 groups of 10. The study involved results in prelims and finals obtained at the Olympic Games in Sydney 2000 (Australia), Athens 2004 (Greece), Beijing 2008 (China), London 2012 (United Kingdom), Rio de Janeiro 2016 (Brazil), and Tokyo 2020 (Japan); and at the World Championships in Melbourne 2007 (Australia), Rome 2009 (Italy), Shanghai 2011 (China), Barcelona 2013 (Spain), and Budapest 2019 and 2022 (Hungary), with total years falling within the range of 2000–2022 (34 results, i.e., 56.7% of all investigated results). The study also involved results in other national or international competitions: European Championships in Budapest 2010; Berlin 2014 (Germany); Glasgow 2018 (United Kingdom); Pan Pacific Championships in Tokyo 2018; Commonwealth Games in Gold Coast 2018 (Australia); Universiade in Belgrade 2009 (Serbia); Kazan 2015 (Russia); Gwangju 2015 (South Korea); National Championships of Australia in Adelaide 2013, 2016 (Australia); National Championships of Great Britain in London 2012; Glasgow 2016, 2019; National Championships of Russia in Kazan 2021 and 2022; National Championships of Netherlands in Eindhoven 2011 (Netherlands); NSW State Open Championships in Sydney 2012; US Olympic Games Trials in Omaha 2012 (USA); US World Championships Team Trials in Greensboro 2022 (USA); Swim Open Stockholm in Stockholm in 2017, 2019 (Sweden); Sette Colli in Rome 2020; TYR Pro Swim Series in Richmond 2019 (USA), with total years falling within the range of 2009–2022 (26 results, i.e., 43.3% of all investigated results). The first group of swimmers (ranked 1–10) was characterized by an age of 22.3 ± 2.63 years (mean ± standard deviation [SD]), and further groups of 10 were aged 20.6 ± 2.50, 22.3 ± 2.16, 22.4 ± 3.53, 21.8 ± 3.01, and 21.1 ± 2.47 years. The age for all the examined swimmers equalled 21.75 ± 2.72 years. All procedures used in the study were approved by the University Bioethics Committee for Scientific Research of the Jerzy Kukuczka Academy of Physical Education in Katowice (No. 8/2018) with the exception of the requirement for the informed consent of participants due to the fact that the study included an analysis of publicly available data.

### 2.2. Statistical Analysis

The normality of the distribution of the tested variables was assessed with the Shapiro–Wilk test. The homoscedasticity and sphericality of data were analysed with Levene’s and Mauchly’s tests, respectively. In the context of the research questions posed, analysis of variance was used to calculate the differences in the splits of the 1500 m swimming race between the sixth-tenth of the best swimmers. Post hoc pairwise comparison analysis was performed with Bonferroni’s and Scheffé’s tests. A mixed-design analysis of variance distinguished an intergroup variable: group, with 6 categories (from one—to sixth-tenth of best swimmers); and an intragroup variable: split time, expressed as a repeated measure in the subsequent splits of the 1500 m distance. The mixed-design analysis of variance employed 2 splits (2 × 750 m), 3 splits (3 × 500 m), 5 splits (5 × 300 m), and 15 splits (15 × 100 m).

The 2 × 6, 3 × 6, 5 × 6, and 15 × 6 analysis plans included the assessment of main effects and interaction effects. The effect size was expressed as the partial eta-squared (η_p_^2^) ratio and interpreted as low (0.01), moderate (0.09), or strong (0.25). A *p*-value of ≤0.05 was assumed as statistically significant. The analyses were performed with the Statistica 13.3 software (TIBCO Software Inc., Palo Alto, CA, USA).

## 3. Results

The results of the analysis of variance are presented in Table 1. One can observe that the main effects of the competitor group order are statistically significant (*p* < 0.001; η_p_^2^ = 0.95), and each subsequent group of ten athletes achieved significantly slower times in 1500 m freestyle swimming. The interaction effect of the competitor group order and the splits of the 1500 m distance for the 2 splits (F_(5, 54)_ = 0.57; *p* = 0.723; η_p_^2^ = 0.05), 3 splits (F_(10, 108)_ = 0.83; *p* = 0.560; η_p_^2^ = 0.07), 5 splits (F_(20, 216)_ = 1.00; *p* = 0.459; η_p_^2^ = 0.09), and 15 splits (F_(70, 756)_ = 1.09; *p* = 0.294; η_p_^2^ = 0.09) turned out to be statistically insignificant. The magnitude of the interaction effects was moderate (η_p_^2^ of 0.05–0.09). The investigation revealed that the successive groups of the best and worst performers over the 1500 m distance did not differ in the distribution of pacing in the subsequent splits in any of the distance division approaches.

The main effect of the 2 × 750 m splits was statistically insignificant (F_(1, 54)_ = 0.00; *p* = 0.999; η_p_^2^ = 0.00). The magnitude of this effect is zero, and no differences were found between the results of the first (444.50 ± 3.75 s) and second (444.50 ± 4.22 s) halves of the 1500 m distance, as demonstrated in Table 2 of the post hoc analysis. The main effects of the 3 × 500 m (F_(2, 108)_ = 27.04; *p* < 0.001; η_p_^2^ = 0.33), 5 × 300 m (F_(4, 216)_ = 66.15; *p* < 0.001; η_p_^2^ = 0.55), and the 15 × 100 m (F_(14, 756)_ = 162.30; *p* < 0.001; η_p_^2^ = 0.75) splits were statistically significant. The effect sizes of these analyses reached very high values (η_p_^2^ of 0.33–0.75). It is worth noting that the more detailed the divisions of the 1500 m distance into splits, the higher the effect size. Multiple comparisons (post hoc analysis showed in Table 2) indicated that the first (295.62 ± 2.82 s) and last (295.50 ± 3.20 s) sections of the 3 × 500 m split were significantly better than the second middle section (297.91 ± 2.45 s).

Moreover, no significant differences were observed between the first and third sections. In the assessment of the differences between the 1500 m distance sections in the 5 × 300 m split, the post hoc analysis (Table 3) revealed that the first (176.31 ± 1.97 s) and fifth (176.28 ± 2.35 s) sections had significantly faster times than the second (178.87 ± 1.42 s), third (178.71 ± 1.51 s), and fourth (178.85 ± 1.70 s) sections.

In addition, the differences between the first and fifth sections and between the second, third, and fourth sections were not statistically significant (Table 4).

**Figure 1:**
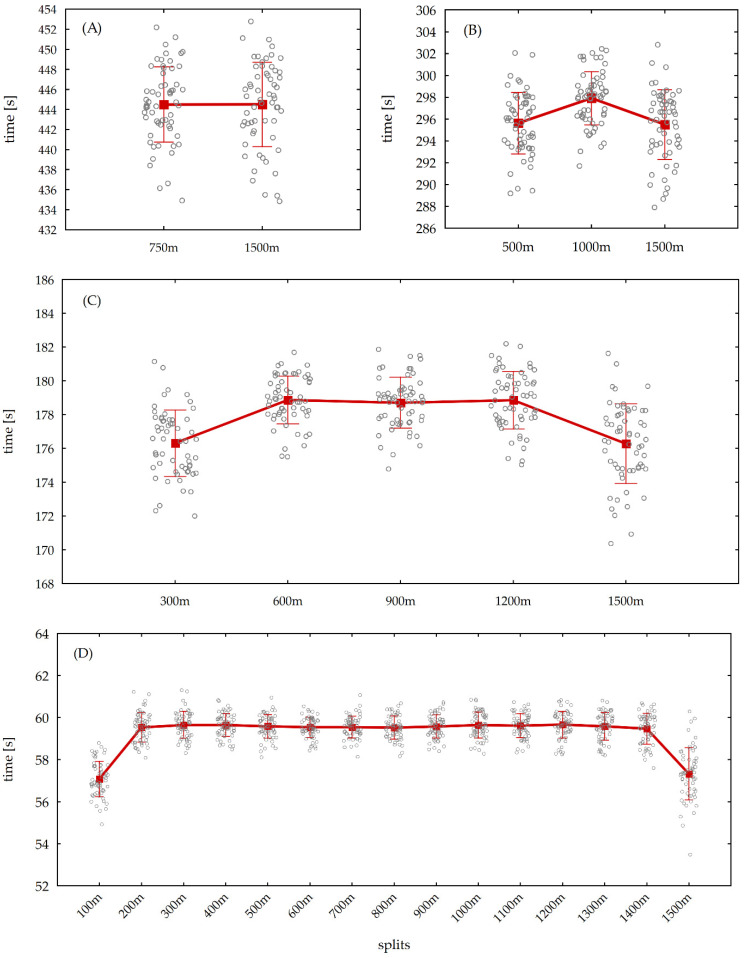
Mean and standard deviation of the (**A**) 750 m, (**B**) 500 m, (**C**) 300 m, and (**D**) 100 m splits in the pace distribution among the 60 all-time best competitors in 1500 m male freestyle long-course (50 m) swimming.

In the 15 × 100 m split, the post hoc analysis demonstrated that the first (57.08 ± 0.84 s) and last (57.33 ± 1.24 s) sections were significantly better than sections 2–14. One should recognize that the first and last sections did not differ significantly in time, but sections 2–14 were also characterized by similar, non-significantly different results. The results obtained in the analysis for the particular splits are depicted in Figure 1.

## 4. Discussion

In the present study, the averaged pace distribution among the 60 all-time best competitors in 1500 m male freestyle long-course (50 m) swimming was investigated. On the basis of the results, the following conclusions can be raised: (a) the swimmers in each of the top-ten groups in the all-time ranking distributed the pace over the distance swum without significant differences, (b) the first and last sections of the distance were covered significantly faster than the middle sections, for which the times did not differ significantly from one another, and (c) the pace distribution model assumes a parabolic pattern.

The first important conclusion of the analysis concerns the comparison of the first and the further ten competitors in the ranking in terms of swimming pace distribution. The analysis revealed that the athletes in the particular groups of ten distributed the pace over the distance swum without significant differences. This means that the best swimmers in the top ten and those in the subsequent group of tens presented a very similar race strategy, although their times differed significantly. Despite the fact that the athletes’ final race times and split times are markedly different, one can clearly notice the same concept of racing and of distributing force over different parts of the race. Lara and Del Coso (2021) reached similar conclusions when examining pacing strategy models among World Championships competitors [18]. They did not report significant differences among athletes ranked higher and lower in the finals. This demonstrates that elite-level competitors are excellently trained in memorizing a set pace and maintaining consistency [19]. They achieve this through experience and appropriately adapted training [20,21,22] to make the most effective use of energy while limiting fatigue in the initial phase [4].

The second conclusion of the analysis refers to the tendency that the first and last sections of the distance were covered significantly faster than the middle sections, for which the times did not differ significantly from one another, regardless of whether the distance was divided into 15, 5, or 3 sections (100 m, 300 m, and 500 m, respectively). The first section, which begins with a leap from the starting block into the water, is significantly faster than the following ones. This may not only be due to the large amount of energy that athletes have at the start of the distance but also to the acceleration provided by the jump and glide after the starter’s command [1,8,23,24]. The final section is characterized by maximum work up to the end of the race, as is allowed by the remaining energy, which can to some extent compensate for the inappropriate pace distribution over the distance [8,24]. The middle part of the distance, which is also the longest, is covered at a very even pace with minor deviations. Swimmers focus on maintaining an even best pace for as long as possible. The pace is slower than at the beginning and the end of the distance, and the decrease in speed is intended to ensure that energy is maintained until the very end of the race [5,25]. According to McGibbon (2018), this ability to sustain the swimming speed throughout the race is key to optimizing performance [4]. López-Belmonte and his colleagues (2022) showed a similar tendency not only during the longest freestyle distance but also during shorter ones, such as 400 and 800 m [26]. The conclusions drawn from the conducted analysis refer to swimming, but a similar trend can also be identified in scientific papers on other sports, such as rowing [27], speed skating [28], running [29], and cycling [30].

The third and final conclusion, partly stemming from the previous one, is that the developed pace distribution model assumes a parabolic pattern. This is consistent with the findings of other researchers who demonstrated that this very pacing strategy was most commonly adopted in long-distance swimming [9,13,24,31]. As discussed earlier, the ability to maintain an even pace over most of the distance is crucial to the outcome. Neuloh with his colleagues (2022) observed that the end-spurt in 1500 m events was crucial to achieving a medal, finding a more pronounced end-spurt in medallists compared to non-medallists [32]. Any modification in the rhythm and pace of swimming can translate into functional and metabolic changes [3,33] and, consequently, affect performance and the final result [34]. The aim of every swimmer is to maintain the highest possible capacity while minimizing fatigue, i.e., performance impairment reducing the ability to produce the required strength or power output [35]. The parabolic model of pace distribution, applied by almost all swimmers in the presented analysis, indicates that sporting success in long-distance swimming is founded on maintaining a fast and, above all, constant pace for as long as possible between the fastest but shortest bursts of speed at the start and finish of the race.

In this study, solely the best results in the history of the competition were considered for analysis. The findings provide a clear message regarding the optimal pace distribution and strategy adopted during a race based on the outstanding performance of athletes who won major titles and set records at the international level. Applying such models by training staff and competitors increases the chances of competition success compared with looking for the appropriate strategy by trial and error. While it is certainly possible to individualize the pacing strategy for a given competitor, this study provides a ready-made and optimal model that has resulted in the most prominent athletic achievements in swimming. The limitations of this study are the results for the 60 swimmers, without divisions into prelims and finals. It will be beneficial to increase the number of swimmers tested and show the difference between swimming in the morning and in the afternoon, because it is during these times of the day that competitions are usually held. In the future, an analysis that would report the differences between results obtained in indoor and outdoor pools and even differences in performances across the hemispheres of Earth or continents is recommended. It remains unclear whether there are discrepancies between these types of swimming, which constitutes a good starting point for developing such analyses and seeking new conclusions.

## 5. Conclusions

Among the all-time best competitors in 1500 m male freestyle long-course swimming, the pacing strategy turned out to be very consistent and exhibited similar trends. Although the ultimate finishing times varied considerably, a uniform strategy for completing the race can be observed: the first and last sections of the distance are covered faster than the middle ones, which are characterized by minor and insignificant split time deviations, and the entire pace distribution model assumes a parabolic pattern.

## Figures and Tables

**Table 1:** Summarized variance analysis of the recorded split times in the 1500 m swimming performance categorized by the number of splits and subsequent groups of swimmers.

Splits	Effect	*F*	*df*	*p*	η_p_^2^
2 × 750 m	Group	218.20	5, 54	<0.001	0.95
	Split time	0.00	1, 54	0.999	0.00
	Group × Split time	0.57	5, 54	0.723	0.05
3 × 500 m	Group	213.30	5, 54	<0.001	0.95
	Split time	27.04	2, 108	<0.001	0.33
	Group × Split time	0.83	10, 108	0.560	0.07
5 × 300 m	Group	216.08	5, 54	<0.001	0.95
	Split time	66.15	4, 216	<0.001	0.55
	Group × Split time	1.00	20, 216	0.459	0.09
15 × 100 m	Group	218.20	5, 54	<0.001	0.95
	Split time	162.30	14, 756	<0.001	0.75
	Group × Split time	1.09	70, 756	0.294	0.09

*F*—result of variance analysis; *df*—degree of freedom; *p*—value; η_p_^2^—effect size expressed as partial eta-squared.

**Table 2:** Summarized post hoc Bonferroni’s and Scheffé’s tests analysis of the 500 m split of the 1500 m swimming performance.

Splits		Time (s)	Bonferroni’s Significance Results		
		*M* ± *SD*	1	2	3
500 m	1	295.62 ± 2.82	–		
1000 m	2	297.91 ± 2.45	<0.001 *	–	
1500 m	3	295.50 ± 3.20	n.s	<0.001 *	–

Note. *N* = 60. * Significant Scheffé’s post hoc test comparison (*p* < 0.05). n.s—not significant.

**Table 3:** Summarized post hoc Bonferroni’s and Scheffé’s tests analysis of the 300 m split of the 1500 m swimming performance.

Splits		Time (s)	Bonferroni’s Significance Results					
		*M* ± *SD*	1	2	3	4	5
300 m	1	176.31 ± 1.97	–					
600 m	2	178.87 ± 1.42	<0.001 *	–				
900 m	3	178.71 ± 1.51	<0.001 *	n.s	–			
1200 m	4	178.85 ± 1.70	<0.001 *	n.s	n.s	–		
1500 m	5	176.28 ± 2.35	n.s	<0.001 *	<0.001 *	<0.001 *	–	

Note. *N* = 60. * Significant Scheffé’s post hoc test comparison (*p* < 0.05). n.s—not significant.

**Table 4:** Summarized post hoc Bonferroni’s and Scheffé’s tests analysis of the 100 m split of the 1500 m swimming performance.

Splits		Time (s)	Bonferroni’s Significance Results	
		*M* ± *SD*	1	2–14
100 m	1	57.08 ± 0.84	–	
200 m	2	59.53 ± 0.70	<0.001 *	–
300 m	3	59.65 ± 0.64	<0.001 *	n.s
400 m	4	59.65 ± 0.54	<0.001 *	n.s
500 m	5	59.58 ± 0.56	<0.001 *	n.s
600 m	6	59.54 ± 0.48	<0.001 *	n.s
700 m	7	59.55 ± 0.52	<0.001 *	n.s
800 m	8	59.53 ± 0.55	<0.001 *	n.s
900 m	9	59.58 ± 0.55	<0.001 *	n.s
1000 m	10	59.65 ± 0.62	<0.001 *	n.s
1100 m	11	59.62 ± 0.57	<0.001 *	n.s
1200 m	12	59.66 ± 0.64	<0.001 *	n.s
1300 m	13	59.59 ± 0.66	<0.001 *	n.s
1400 m	14	59.47 ± 0.73	<0.001 *	n.s
1500 m	15	57.33 ± 1.24	n.s	<0.001 *

Note. *N* = 60. * Significant Scheffé’s post hoc test comparison (*p* < 0.05). n.s—not significant.

## Data Availability

Data available in a publicly accessible websites.
